# Factors Associated with Veteran Self-Reported Use of Digital Health Devices

**DOI:** 10.1007/s11606-023-08479-8

**Published:** 2024-01-22

**Authors:** Stephanie A. Robinson, Stephanie L. Shimada, Mark S. Zocchi, Bella Etingen, Bridget Smith, Nicholas McMahon, Sarah L. Cutrona, Julie S. Harmon, Nancy R. Wilck, Timothy P. Hogan

**Affiliations:** 1 eHealth Partnered Evaluation Initiative, Veterans Affairs Bedford Healthcare System, 200 Springs Rd., Bldg. 70 Room 263, Bedford, MA 01730 USA; 2 Center for Healthcare Organization and Implementation Research (CHOIR), VA Bedford Healthcare System, Bedford, MA USA; 3 The Pulmonary Center, Boston University School of Medicine, Boston, MA USA; 4 Department of Health Law, Policy, & Management, Boston University School of Public Health, Boston, MA USA; 5 Division of Health Informatics and Implementation Science, Department of Population and Quantitative Health Sciences, University of Massachusetts Chan Medical School, Worcester, MA USA; 6 Heller School for Social Policy and Management, Brandeis University, Waltham, MA USA; 7 Center of Innovation for Complex Chronic Healthcare, Hines Veterans Affairs Hospital, Hines, IL USA; 8 Northwestern University Feinberg School of Medicine, Chicago, IL USA; 9 Office of Connected Care, Veterans Health Administration, US Department of Veterans Affairs, Washington, DC USA; 10 Peter O‘Donnell Jr School of Public Health, University of Texas Southwestern Medical Center, Dallas, TX USA

**Keywords:** wearable, eHealth, telemedicine, health technology adoption, self-management

## Abstract

**Background:**

Digital health devices (DHDs), technologies designed to gather, monitor, and sometimes share data about health-related behaviors or symptoms, can support the prevention or management of chronic conditions. DHDs range in complexity and utility, from tracking lifestyle behaviors (e.g., pedometer) to more sophisticated biometric data collection for disease self-management (e.g., glucometers). Despite these positive health benefits, supporting adoption and sustained use of DHDs remains a challenge.

**Objective:**

This analysis examined the prevalence of, and factors associated with, DHD use within the Veterans Health Administration (VHA).

**Design:**

National survey.

**Participants:**

Veterans who receive VHA care and are active secure messaging users.

**Main Measures:**

Demographics, access to technology, perceptions of using health technologies, and use of lifestyle monitoring and self-management DHDs.

**Results:**

Among respondents, 87% were current or past users of at least one DHD, and 58% were provided a DHD by VHA. Respondents 65 + years were less likely to use a lifestyle monitoring device (AOR 0.57, 95% CI [0.39, 0.81], *P* = .002), but more likely to use a self-management device (AOR 1.69, 95% [1.10, 2.59], *P* = *.*016). Smartphone owners were more likely to use a lifestyle monitoring device (AOR 2.60, 95% CI [1.42, 4.75], *P* = .002) and a self-management device (AOR 1.83, 95% CI [1.04, 3.23], *P* = .037).

**Conclusions:**

The current analysis describes the types of DHDs that are being adopted by Veterans and factors associated with their adoption. Results suggest that various factors influence adoption, including age, access to technology, and health status, and that these relationships may differ based on the functionalities of the device. VHA provision of devices was frequent among device users. Providing Veterans with DHDs and the training needed to use them may be important factors in facilitating device adoption. Taken together, this knowledge can inform future implementation efforts, and next steps to support patient-team decision making about DHD use.

## INTRODUCTION

Digital health devices (DHDs) are designed to gather, track, monitor, and sometimes share data about health-related behaviors or symptoms. The growing development and use of DHDs represents a technological revolution in healthcare, allowing patients and their care teams to continuously monitor health behaviors and outcomes outside of the clinical visit.^1, 2^ The aging US population and increased prevalence of patients living with multiple chronic conditions^3^ underscores the need to better engage patients in their own health in an effort to ameliorate health care resource burden.^4^ The Veterans Health Administration (VHA) Office of Connected Care is committed to improving health care through technology by engaging Veterans and their clinical teams outside of episodic health care visits, which can in part be supported by patient use of DHDs.^5^

DHDs and their associated data range in functions, from providing feedback to users to help them understand their health (i.e., lifestyle monitoring devices) to supporting preventative and self-management behaviors (i.e., self-management devices), thereby improving how patients and their clinical teams prevent and/or manage chronic conditions.^6, 7^ This functionality is especially relevant for Veterans, who face disproportionate rates of chronic disease compared to the general US adult population.^8^

Despite the potential benefits of DHDs, supporting their adoption remains a challenge. Most literature describes consumer *intention* to adopt DHDs,^9, 10^ rather than actual adoption. The objective of the current analysis was to examine use and perceptions of different types of DHDs among Veterans who receive healthcare within the VHA, and to identify factors associated with use of DHDs.

## METHODS

### Design

The Veterans Engagement with Technology Collaborative (VET-C) cohort was initiated in 2017 to help inform quality improvement and implementation efforts focused on virtual care technology implementation and use in VHA. ^11^ It includes longitudinal survey data collected at three time-points from a nationwide sample of Veterans who are known technology users, coupled with demographic and health information from VHA administrative data. Surveys collected data on Veteran perceptions of VHA healthcare, technology ownership and use, and preferences for using technology to support their health. VET-C was supported by VHA’s Quality Enhancement Research Initiative (QUERI) Program and the VHA Office of Connected Care. The Office of Connected Care’s mission is to deliver high-quality, Veteran-centered care, optimize individual and population health, advance health care that is personalized and proactive, and enhance the health care experience through virtual modalities of care.^12^ This work was designated as a program evaluation for quality improvement purposes by the affiliated institutional review boards (VHA Handbook 1058.05).

### Sample

The VET-C cohort was sampled from 15 geographically dispersed facilities (see Appendix).^11^ Veterans who were active users of health-related technologies were purposefully sampled as they were thought to be most willing to make a long-term commitment to providing feedback on VHA virtual care technologies, including DHDs and mobile health applications. Therefore, mobile phone ownership and secure messaging use (i.e., having sent ≥ five messages in the year prior to cohort recruitment) were sampling inclusion criteria. Approximately 52% of national VHA users have access to use secure messaging through the patient portal, and 27% of national VHA users are active users of secure messaging.^13^ Secure messaging use was used as a proxy for Veteran openness to using new technologies, as well as their use of other VHA patient-facing technologies more broadly.

### Data Collection Procedures

Survey data were collected from the VET-C cohort at three time points: 2017–2018, 2019–2020, and 2021. Procedures for these first two rounds of data collection are described in previous publications.^11, 14, 15^ Veterans who responded to the first two rounds were invited to complete a third survey between May and December 2021. Veterans were mailed a hard copy of this survey; non-responders were mailed an additional copy four weeks later. A total of 1,373 Veterans were invited to participate in this third survey round; the denominator was adjusted to 1,358 to reflect five Veterans who were deceased and 10 surveys which were returned as undeliverable. We received responses from 858 Veterans (858/1,358, 63.2% response rate). Data presented in the current analysis was largely collected in the third round of VET-C survey administration, although some demographic data (i.e., age, race, ethnicity, gender, educational attainment) was gathered in the first round of survey administration (detailed below). In addition, survey data were supplemented with electronic health record data from the VHA Corporate Data Warehouse, as appropriate.

### Measures

#### Digital Health Device Use

Surveys asked Veterans to report their use of nine DHDs, which were then categorized based on The National Institute for Health and Care Excellence^16^ classification of health technologies across three evidence-based tiers. The current analysis focuses on the second and third tiers, which describe DHDs that help users to (1) understand healthy living and illnesses through informing and simple monitoring (i.e., *lifestyle monitoring devices*) and (2) prevent and manage diseases (i.e., *self-management devices*).^16^ The team reached consensus on the appropriate classification for each device based on this previously published criteria.^16^ Among the nine DHDs represented on the VET-C survey, three were categorized as lifestyle monitoring DHDs: Fitbit, smartwatch, and digital pedometer. As these three devices typically offer similar functionality, we collapsed them into one category. The remaining six DHDs were categorized as self-management DHDs: blood pressure monitor, electrocardiogram (EKG/ECG) monitor, glucometer, asthma inhaler, pulse oximeter, and spirometer. Participants were asked to indicate if they: currently use the device, used the device in the past but no longer use it, or if they have never used it. Participants also indicated if VHA provided them with any of the above devices.

#### Covariates

Data collected from the VHA Corporate Data Warehouse included rurality of residence and Hierarchical Condition Category (HCC)^17^ scores. HCC – a measure of comorbidity – accounts for age, gender, medical diagnoses using ICD-10 codes, and eligibility for Medicare and Medicaid services.^17^ Normalized to 1.0, HCC scores < 1.0 are considered scores of relatively healthy individuals.^18^

Respondents reported factors associated with their physical health, health care use, (i.e., whether they usually receive care within or outside of VHA), marital status, and socioeconomic status (SES; difficulty paying for basics like food or heating/cooling). Veterans were also asked about their access to technology (i.e., “Do you own or have easy access to a: computer, tablet, smartphone?”).

### Analyses

We classified participants into DHD “users” and “non-users.” Users indicated current or past use of a DHD; non-users indicated no current or previous use of a DHD. Some respondents skipped all questions related to DHDs; thus, we could not determine whether these respondents were device users. In addition, respondents were not included if they skipped questions used as covariates in the multivariable models. The final analytic sample included 846 Veterans. Univariate analyses characterized the sample. We modeled three multiple logistic regressions to assess factors associated with three outcomes: 1) any DHD use, 2) lifestyle monitoring DHD use, and 3) self-management DHD use. Adjusted odds ratios (AORs) and 95% confidence intervals were calculated to measure the association between each factor and each outcome, after controlling for other variables in the model. AORs with 95% confidence intervals that did not include 1.00 were considered statistically significant.

## RESULTS

Table 1 presents demographic characteristics. Most were ≥ 65 years of age (71.7%, *n* = 607), male (87.5%, *n* = 740), white (88.9%, *n* = 752), non-Hispanic (96.8%, *n* = 819), married (68.3%, *n* = 578), and non-rural (85.1%, *n* = 720). Most reported that paying for basic necessities was “not very hard” (68.8%, *n* = 582) and education beyond high school (87%, *n* = 736), with a large portion having a master’s, professional, or doctoral degree (48.6%, *n* = 411). Most (86.6%, *n* = 733) indicated either current or previous DHD use (i.e., DHD users). More than half (57.8%, *n* = 489) reported that VHA provided them with at least one DHD. Among those who received a DHD from VHA, only 3.5% (*n* = 17) did not report any DHD use. Lifestyle monitoring DHD use was reported by 40.8% (*n* = 345) and self-management DHD use was reported by 79.4% (*n* = 672). Among self-management DHD users (*n* = 672), digital blood pressure monitors were most frequently reported (91.4%, *n* = 614; Table 2).

**Table 1:** Survey Respondent Characteristics (*N* = 846)

	*n*	%
Age 65 and older	607	71.7
Gender		
Female	106	12.5
Male	740	87.5
Race		
White	752	88.9
Black	55	6.5
Other	39	4.6
Hispanic ethnicity		
No	819	96.8
Yes	27	3.2
Marital status		
Married or Civil Union	578	68.3
Not married	240	28.4
Unknown	28	3.3
Rural residence		
No	720	85.1
Yes	126	14.9
Difficulty paying for basic necessities		
Somewhat/Hard/Very hard	186	22.0
Not very hard	582	68.8
Don't know	78	9.2
Educational level		
High school graduate or less	104	12.3
Some college/Bachelor's degree	325	38.4
Master’s/Professional/Doctoral	411	48.6
Declined to answer	6	0.7
Location of care		
Mostly at the VHA	609	72.0
Mostly outside VHA	58	6.9
About half in VHA, half outside VHA	179	21.2
Self-reported physical health		
Excellent/very good	226	26.7
Good	365	43.1
Fair/poor	255	30.1
I own or have easy access to a…		
Computer	785	92.8
Tablet	446	52.7
Smartphone	756	89.4
Device User		
No	113	13.4
Yes	733	86.6
VHA Provided Device		
No	357	42.2
Yes	489	57.8
Atrial fibrillation	129	15.3
Asthma	201	23.8
CKD	254	30.1
COPD	156	18.5
Depression	319	37.8
Diabetes	371	43.9
Ischemic heart disease	252	29.8
Hypertension	660	78.1
HCC Comorbidity Score, 5 year, mean (SD)	1.66	(1.3)

*VHA*, Veterans Health Administration; *HCC*, Hierarchical Condition Category; *SD*, Standard Deviation

**Table 2:** Type and Frequency of Digital Health Device Use

	Any Use	
	*n*	%
Lifestyle Monitoring Devices	345	40.8
Self-Management Devices		
Digital blood pressure monitor	614	85.5
Digital glucometer	287	42.7
Digital pulse oximeter	197	29.3
Digital asthma inhaler	84	12.5
Digital electrocardiogram monitor	63	9.4
Digital spirometer	28	4.2

### Factors associated with Digital Health Device Use

Results from the three multiple logistic regressions examining: (1) any DHD use, (2) lifestyle monitoring DHD use, and (3) self-management DHD use are in Table 3. When we examined *any DHD use*, Hispanic respondents had lower odds of being a DHD user compared to non-Hispanic respondents (AOR 0.22, 95% CI [0.06, 0.76], *P* = 0.017). Veterans with a master’s, professional, or doctoral degree had higher odds of being a device user compared to those with a high school education or less (AOR 2.07, 95% CI [1.06, 4.03], *P* = 0.033. Compared to those who reported receiving most of their care at VHA, those who reported receiving about half of their care outside VHA had greater odds of being a DHD user (AOR 1.88, 95% CI [1.03, 3.448], *P* = 0.040). Those who self-reported fair/poor physical health (compared to excellent/very good health) had higher odds of being a DHD user (AOR 2.58, 95% CI [1.33, 5.00], *P* = *0.005).* Similarly, a higher HCC score (i.e., worse health) was associated with greater odds of being a DHD user (AOR 1.42, 95% CI [1.11, 1.82], *P* = 0.005). Further, respondents had higher odds of being a DHD user if they reported ownership or easy access to a tablet (AOR 1.81, 95% CI [1.16, 2.84], *P* = 0.009), or smartphone (AOR 2.13, 95% CI [1.17, 3.89], *P* = 0.014) compared to those who did not. Age, gender, race, marital status, rurality, SES, and computer access were not significantly associated with overall DHD use.

**Table 3:** Multivariate Logistic Regressions of Factors associated with Digital Health Device Use (*N* = 841)

	All			Lifestyle Monitoring			Self-Management		
	AOR	[95% CI]	*P*	AOR	[95% CI]	*P*	AOR	[95% CI]	*P*
Age 65 and older	1.22	[0.73, 2.03]	0.457	0.57	[0.39, 0.81]	0.002	1.69	[1.10, 2.59]	0.016
Gender (ref = Female)									
Male	1.62	[0.88, 3.00]	0.121	0.90	[0.56, 1.46]	0.680	2.50	[1.49, 4.19]	0.001
Race (ref = White)									
Black	0.63	[0.28, 1.41]	0.262	1.73	[0.92, 3.25]	0.090	0.76	[0.38, 1.53]	0.449
Other	1.88	[0.51, 6.86]	0.339	1.98	[0.84, 4.67]	0.120	1.20	[0.43, 3.36]	0.726
Hispanic (ref = Not Hispanic)	0.22	[0.06, 0.76]	0.017	0.39	[0.14, 1.09]	0.073	0.44	[0.14, 1.35]	0.152
Marital status (ref = Not married)									
Married	0.77	[0.47, 1.25]	0.293	1.03	[0.72, 1.46]	0.881	0.67	[0.44, 1.03]	0.069
Unknown	2.40	[0.47, 12.18]	0.290	1.15	[0.46, 2.87]	0.770	2.41	[0.60, 9.72]	0.217
Rural residence (ref = not rural)									
Rural	1.03	[0.57, 1.87]	0.913	0.82	[0.54, 1.27]	0.377	1.01	[0.61, 1.69]	0.965
Difficulty paying for basic necessities (ref = Somewhat/Hard/Very Hard)									
Not very hard	0.63	[0.35, 1.14]	0.125	1.30	[0.89, 1.91]	0.178	0.81	[0.50, 1.32]	0.396
Don't know	0.58	[0.23, 1.45]	0.247	1.50	[0.79, 2.85]	0.211	0.62	[0.28, 1.35]	0.224
Educational level (ref = ≤ High school)									
Some college /Bachelor's degree	1.38	[0.71, 2.66]	0.338	1.14	[0.68, 1.91]	0.625	1.48	[0.81, 2.69]	0.198
Master’s/Professional/ Doctoral	2.07	[1.06, 4.03]	0.033	1.76	[1.06, 2.94]	0.030	1.64	[0.91, 2.97]	0.102
Declined to answer	0.21	[0.03, 1.44]	0.113	1.28	[0.21, 7.63]	0.790	0.36	[0.05, 2.38]	0.288
Location of care (ref = Mostly at the VHA)									
Mostly outside VHA	1.18	[0.49, 2.82]	0.713	0.69	[0.38, 1.27]	0.231	1.11	[0.54, 2.31]	0.774
About half in VHA, half outside VHA	1.88	[1.03, 3.44]	0.040	1.22	[0.85, 1.77]	0.283	1.26	[0.79, 2.00]	0.331
Self-reported health (ref = Excellent/very good)									
Good	1.41	[0.87, 2.28]	0.168	1.40	[0.96, 2.03]	0.077	1.76	[1.15, 2.68]	0.009
Fair/poor	2.58	[1.33, 5.00]	0.005	1.22	[0.79, 1.88]	0.364	2.06	[1.21, 3.52]	0.008
Computer access	2.10	[1.00, 4.44]	0.051	1.06	[0.58, 1.95]	0.849	1.57	[0.79, 3.13]	0.196
Tablet access	1.81	[1.16, 2.84]	0.009	2.34	[1.72, 3.19]	0.000	1.22	[0.83, 1.78]	0.307
Smartphone access	2.13	[1.17, 3.89]	0.014	2.60	[1.42, 4.75]	0.002	1.83	[1.04, 3.23]	0.037
HCC Score, 5 year	1.42	[1.11, 1.82]	0.005	0.97	[0.85, 1.11]	0.671	1.67	[1.33, 2.10]	0.000

*VHA*, Veterans Affairs; *AOR*, Adjusted Odds Ratio (adjusted for other predictor variables in the model); *CI*; Confidence Interval; *HCC*, Hierarchical Condition Category

Regarding *lifestyle monitoring DHD use*, respondents who were ≥ 65 years (AOR 0.57, 95% CI [0.39, 0.81], *P* = 0.002) had lower odds of lifestyle monitoring DHD use compared those who were below 65 years of age. Veterans with a master’s, professional, or doctoral degree had higher odds of using a lifestyle monitoring DHD compared to those with a high school education or less (AOR 1.76, 95% CI [1.06, 2.94], *P* = 0.030. Those who reported ownership or easy access to a tablet (AOR 2.34, 95% CI [1.72, 3.19], *P* < 0.001), or smartphone (AOR 2.60, 95% CI [1.42, 4.75], *P* = 0.002) had higher odds of lifestyle monitoring DHD use compared to those who did not report ownership or easy access. Gender, race, ethnicity, marital status, rurality, SES, location of care, self-reported physical health, ownership or easy access to a computer, and HCC score were not significantly associated with lifestyle monitoring DHD use.

When we examined *self-management DHD use*, respondents who were ≥ 65 years (AOR 1.69, 95% CI [1.10, 2.59], *P* = 0.016), or male (AOR 2.50, 95% CI [1.49, 4.19], *P* = 0.001) had higher odds of use. Compared to respondents who self-reported excellent/very good health, those with good health (AOR 1.76, 95% CI [1.15, 2.68], *P* = 0.009) or fair/poor health (AOR 2.06, 95% CI [1.21, 3.52], *P* = 0.008) had higher odds of using a self-management DHD, as did those who had higher HCC scores (AOR 1.67, 95% CI [1.33, 2.10], *P* < 0.001). Respondents who owned or had easy access to a smartphone (AOR 1.83, 95% CI [1.04, 3.23], *P* = 0.037) also had higher odds of using a self-management DHD. Race, ethnicity, marital status, rurality, SES, education, location of care, and computer or tablet ownership/access were not significantly associated with self-management DHD use.

Table 4 displays frequencies and comparisons of DHD use by the most prevalent chronic conditions in the sample. There was a significantly smaller proportion of lifestyle monitoring DHD users (*n* = 87, 25.3%) compared to non-users (*n* = 165, 33.1%) among respondents with ischemic heart disease; *P* = 0.017. There were no other significant differences in lifestyle monitoring DHD use across the examined chronic conditions. Significantly greater proportions of self-management DHD users compared to non-users had atrial fibrillation, diabetes, chronic kidney disease, ischemic heart disease, and hypertension (*P*s < *0.001*). There were no significant differences in self-management DHD use among respondents with asthma or COPD.

**Table 4:** Prevalence of Device use by Chronic Condition

	Lifestyle Monitoring					Self-Management				
	User (*n* = 345)		Non-user (*n* = 501)		Chi-squared	User (*n* = 671)		Non-user (*n* = 175)		Chi-squared
	*n*	%	*n*	%	*P*	*n*	%	*n*	%	*P*
Asthma	77	22.4	124	24.8	0.427	163	24.3	38	21.8	.498
Atrial fibrillation	55	16.0	74	14.8	0.629	119	17.7	10	5.7	< .000
Diabetes	148	43.0	223	44.5	0.669	340	50.7	31	17.8	< .000
Chronic kidney disease	104	30.2	150	29.9	0.927	234	34.9	20	11.5	< .000
Chronic obstructive pulmonary disease	59	17.2	97	19.4	0.416	131	19.5	25	14.4	.118
Ischemic heart disease	87	25.3	165	32.9	0.017	228	34.0	24	13.8	< .000
Hypertension	260	75.6	400	79.8	0.141	586	87.3	74	42.5	< .000

## DISCUSSION

Age, ethnicity, education, access to technology, and health were associated with DHD use in this cohort of US Veterans. Some associations differed by DHD functionality, extending existing knowledge on DHD use which has mostly focused on general DHD adoption. Notably, recent work found younger age was associated with a greater likelihood of general DHD use.^19^ The current evaluation found that this direction varied by device functionality; older adults were less likely to use a lifestyle monitoring DHD, but more likely to use a self-management DHD. Older adults are more likely to have a chronic disease^20^ and therefore may be more likely to be recommended a self-management DHD by their clinical team. However, the association between age and self-management DHD use was still significant after adjusting for comorbidities using HCC scores. Older adults’ lower likelihood to adopt a lifestyle monitoring DHD may also be attributed to them feeling less familiar with lifestyle monitoring DHDs or the benefits they could provide. Lifestyle monitoring is associated with enhanced disease management, and a reduced risk for chronic diseases and mortality.^21–23^ Despite the benefit lifestyle monitoring DHDs could have on clinical outcomes related to many chronic diseases, respondents with specific chronic conditions were not more likely to use a lifestyle monitoring DHD. It is important to raise clinical team members’ awareness of how lifestyle monitoring DHDs can yield data to support clinical or self-management of chronic conditions. As digital literacy remains a significant barrier to device adoption,^24^ investing in education and training for patients who are more hesitant to adopt DHDs will also be needed.

Significantly more survey respondents with diabetes, chronic kidney disease, ischemic heart disease, hypertension, or atrial fibrillation were users of a self-management DHD. Chronic respiratory diseases (asthma and COPD) were not associated with self-management DHD use. It may be that certain self-management DHDs (e.g., blood pressure cuffs, glucometers) have historically been more available compared to more recent commercialization of DHDs for chronic respiratory diseases. Given the high prevalence and burden of chronic respiratory disease, particularly in Veterans,^25^ future initiatives may look to targeting adoption efforts for devices specific to respiratory monitoring (e.g., digital pulse oximeters, digital asthma inhalers).^26^ This could include engaging healthcare systems to purchase DHDs for respiratory monitoring. One of the most prominent barriers to adoption of DHDs is their cost.^27^ In this sample, most device users reported being provided a device by VHA. VHA policy allows many different DHDs to be covered; however, access requires care teams and Veterans to be effectively informed of their availability.^28^ As provision of devices may reduce barriers to their use, it is important that VHA care team members and Veterans are educated about their availability as part of VHA benefits.

We did not find significant differences in DHD use by race, rurality, or SES. Hispanic respondents were less likely to be DHD users, but the sample of Hispanic Veterans within our analytic cohort was too small to find significant differences when examining lifestyle monitoring DHD use and self-management DHD use separately. Previous literature has documented disparities in health-related technology use based on SES (i.e., “the digital divide”). It is therefore noteworthy that our analysis did not identify such disparities among this sample. One possible explanation is that our sample was highly educated, particularly in comparison to the overall population of Veterans^29^ and VHA users.^30, 31^ Differences in education and current health status may partially mediate the racial and ethnic disparities commonly seen in technology use.^32^

Smartphone owners were more likely to use both lifestyle monitoring DHDs and self-management DHDs. This is a promising indicator for the trajectory of DHD adoption, as smartphone ownership is increasing annually.^33^ As of 2021, 85% of Americans reported owning a smartphone,^33^ a number similar in magnitude to our sample (90%) and to other broader Veteran populations (81.5%).^34^ Programs that support technology access may also support DHD adoption. For example, VHA’s Offices of Connected Care and Rural Health successfully supported tablet use by distributing tablets to Veterans experiencing barriers to in-person access, with the intention of supporting clinical video visits.^35^ In the current analysis, tablet ownership was associated with lifestyle monitoring DHD use, but not self-management DHD use, and was nearly 40% lower than that of smartphones. This number is comparable to that seen in larger, nationally representative studies.^33^ It is possible that those who own such technologies feel more comfortable with technology and are more likely to use a device. As use of one technology often generates use of others,^36^ any initiatives to support technology ownership will likely further support DHD adoption.

### Limitations and Future Directions

The current analysis describes what types of devices are being adopted among Veterans. While DHDs can enhance health outcomes, not all are necessary for all patients. Additional work is needed to understand optimal applications of DHDs and corresponding dose/response relationships. The current work can inform future implementation efforts and evaluations of the effectiveness of DHD adoption. Additionally, this analysis is limited to patient characteristics. Future work would benefit from integrating healthcare system and healthcare team perspectives to more fully account for the range of possible barriers and facilitators to DHD adoption. Future work would also benefit from better understanding the directionality of these associations. This analysis focused on DHD use but did not capture DHD ownership or other barriers to use. Future work should also explore device ownership, as patients who own but do not use a DHD likely face unique barriers to adoption. Additionally, self-reported DHD use may not accurately reflect actual DHD use. We are also unable to distill respondents’ use of multifunctional devices, or how, if at all, patient-generated data is being communicated back to the care team.

This sample was purposefully recruited to assist quality improvement and implementation efforts focused on virtual care technologies, and as such, was comprised of active users of VHA’s patient portal. Users of VHA’s patient portal are often more educated, younger, and have higher income than the general Veteran population.^37, 38^ In addition, as is typical with the overall Veteran population, our sample was mostly male, and therefore may not be representative of non-Veterans within the general US population. Finally, we are unable to assess the temporal impact of surveying Veterans among this cohort during the COVID-19 pandemic.

## Conclusion

DHDs have the potential to support health promotion and disease management and it is important to identify factors that are associated with device use to support future engagement efforts. Older age was associated with greater odds of self-management DHD adoption, but lower odds of lifestyle monitoring DHD adoption. Additionally, provision of DHDs and access to technology were associated with device adoption. Individuals living with a chronic disease were more likely to adopt a device for self-management, though there remain opportunities to support respiratory disease management and adoption for lifestyle monitoring.

## Appendix

**Table 5:** Location of Respondents and Rurality

Facility (Station Number, Location)	Frequency	Percent Rural
402, Togus, Maine	73	43.8
518, Bedford, Massachusetts	50	4.0
521, Alabama	52	9.6
523, Boston, Massachusetts	92	0.0
541, Cleveland-Wade Park, Ohio	56	5.4
558, Durham, North Carolina	68	16.2
580, Houston Texas	60	6.7
631, North Hampton, Massachusetts	36	5.6
636, Nebraska, W Iowa	112	25.9
656, St. Cloud, Minnesota	44	40.9
660, Salt Lake City, Utah	57	14.0
662, San Francisco. CA	47	14.9
673, Tampa, FL	1	0.0
688, D.C., Maryland, Virginia	56	0.0
691, W. Los Angeles, CA	26	0.0
Missing	16	31.3
Total	846	14.9
